# Learning-Augmented MPC for Autonomous Vehicle Path Tracking via Ensemble Residual Dynamics Learning

**DOI:** 10.3390/s26010340

**Published:** 2026-01-05

**Authors:** Lu Xiong, Ming Liu, Zhihao Xie, Bo Leng, Yuanjian Zhang

**Affiliations:** School of Automotive Studies, Tongji University, 4800 Cao’an Highway, Jiading District, Shanghai 201804, China; xiong_lu@tongji.edu.cn (L.X.);

**Keywords:** autonomous vehicle, path tracking, model predictive control, ensemble learning

## Abstract

Accurate vehicle dynamics modeling is essential for path tracking control, especially under sharp-curvature or rapidly changing conditions where nonlinear and time-varying behaviors introduce significant discrepancies between the nominal model and real vehicle responses, ultimately degrading the performance of traditional Model Predictive Control (MPC). To address this challenge, this paper proposes a learning-augmented MPC framework that incorporates an ensemble learning-based Data-Driven Dynamics Refinement (DDR) Model to enhance predictive accuracy and control robustness. The DDR Model complements nominal vehicle dynamics by capturing complex behaviors that are difficult to represent analytically. An ensemble of independently trained neural predictors is employed to improve generalization performance and provide stable refinement across diverse driving conditions. Furthermore, a feature-driven activation mechanism is designed to selectively apply refinement only when pronounced nonlinear behaviors arise, thereby reducing unnecessary computational burden. High-fidelity simulation studies validate the effectiveness of the proposed method. In single- and double-lane-change scenarios, the refined dynamics reduce maximum lateral deviation by approximately 6 cm and 4 cm, and decrease the maximum vehicle heading error by 0.02 rad and 0.015 rad, respectively, demonstrating significant improvements in tracking accuracy and robustness.

## 1. Introduction

### 1.1. Motivation

The evolution of autonomous driving technologies is driving significant progress in vehicle motion control techniques [1,2]. MPC, owing to its well-recognized capability to handle multiple constraints and generate optimal control sequences, has been widely adopted for autonomous vehicle motion control. Within the theoretical framework of MPC, the controller performs multi-step predictions of the dynamic system states based on a predictive model, which constitutes the core of the control architecture. The accuracy of these predictions directly determines the control performance: inaccurate models introduce prediction errors that can significantly degrade trajectory tracking and stability, whereas precise models enable robust control and reliable vehicle motion. Consequently, developing an accurate and reliable predictive model is essential for achieving high-performance vehicle motion control, particularly under complex and time-varying driving conditions.

Nevertheless, achieving accurate vehicle dynamics modeling remains highly challenging, as the system consists of multiple strongly coupled subsystems with intricate interdependencies [3]. A key challenge lies in tire–road interaction, which exhibits pronounced nonlinearities and uncertainties that are difficult to capture using conventional mathematical models. Consequently, existing vehicle models face a fundamental trade-off: simplified kinematic or low-order dynamic models ensure computational efficiency but fail to reproduce critical nonlinear behaviors such as tire saturation, load transfer, and varying road conditions; in contrast, high-fidelity models provide improved accuracy but are computationally prohibitive for real-time applications. Therefore, reconciling real-time feasibility with high modeling accuracy remains a central challenge in the design of MPC-based motion control systems for autonomous vehicles. These limitations suggest that physics-based models alone are insufficient for representing all dynamic effects. However, instead of replacing the nominal model, a more practical approach is to retain the analytical model as the structural foundation and use data-driven learning to specifically capture the modeling error, i.e., the unmodeled and time-varying dynamics.

### 1.2. Literature Review

Many scholars have proposed various physical models to characterize vehicle dynamics. Some studies [4,5,6] have employed simplified kinematic models in the design of MPC. However, kinematic models primarily focus on the geometric relationships of vehicle motion and fail to accurately capture the vehicle’s dynamic characteristics. Building upon this topic, subsequent research [7,8,9] has proposed a single-track dynamic model that incorporates tire cornering characteristics, providing a more accurate representation of vehicle dynamics. Kabzan et al. [10] proposed a scaling factor-based approach that integrates the single-track dynamic model with the kinematic model, thereby extending the applicability of the fused model and significantly enhancing its accuracy across a broader operational range. However, due to inherent characteristics such as axle load transfer and adherence ellipse constraints during vehicle motion, tire models that assume fixed cornering stiffness exhibit significantly reduced accuracy under varying operating conditions.

In vehicle dynamics research, many scholars have focused on accurately characterizing the interaction forces between tires and the road surface, among which cornering stiffness plays a critical role in lateral and yaw dynamics. However, cornering stiffness is not a fixed value; it is influenced by various factors such as tire construction, inflation pressure, normal ground force, longitudinal tire force, wheel camber angle, and tire wear. Consequently, vehicle models that assume a fixed cornering stiffness struggle to accurately capture lateral and yaw behaviors.

Some studies [11,12] have employed the Recursive Least Squares (RLS) algorithm to enable real-time estimation of tire cornering stiffness for trajectory tracking control. Chu et al. [13] employed Unscented Kalman Filtering (UKF) to estimate cornering stiffness, dynamically correcting the vehicle dynamics model with the estimated results, thereby significantly enhancing both model accuracy and adaptability to varying driving conditions. Additionally, Zhang et al. [14] introduced a Square-Root Cubature Kalman Filtering (SCKF)-based approach for tire lateral force observation. By computing the deviation between observed values and predictions from a linear tire model, a cornering stiffness correction factor is incorporated to enable adaptive adjustment of tire cornering stiffness. While cornering stiffness correction enhances model accuracy, it compensates only for a subset of the modeling errors in vehicle dynamics.

In addition, in traditional control frameworks, Active Disturbance Rejection Control (ADRC) and other model-free or disturbance-estimation-based techniques are often employed to compensate for unknown disturbances and enhance system robustness [15,16]. For example, Wang et al. [17] proposed an ultra-local model predictive control (ULMPC) approach, which replaces an explicit system model with an ultra-local representation and estimates aggregated dynamics online, enabling model-free predictive control for automated vehicle trajectory tracking. Xie et al. [18] developed a disturbance rejection MPC (DRMPC) framework for input-affine nonlinear systems, where a disturbance observer actively compensates matched disturbances, while residual uncertainties are handled through constraint tightening and terminal design to ensure robustness and stability. More recently, Vázquez-Cruz et al. [19] combined MPC with ADRC principles by estimating a lumped total disturbance via an extended state observer and incorporating it into both feedforward compensation and constrained MPC optimization, achieving computationally efficient and robust control under limited computational resources. However, despite their effectiveness in disturbance rejection, these approaches typically lump complex uncertainties into low-dimensional disturbance terms and therefore remain insufficient for capturing the high-dimensional, strongly nonlinear, and operating-condition-dependent modeling errors inherent in vehicle dynamics.

In recent years, data-driven approaches have garnered widespread attention due to their powerful fitting capabilities. These methods have been introduced into vehicle dynamics modeling, where the vehicle model is typically decomposed into a standard dynamics model and a residual dynamics model. Some studies [10,20,21] have employed Gaussian Process Regression (GPR) to fit the residual model, capturing the discrepancy between the single-track dynamics model and actual vehicle dynamics. To enhance the computational efficiency of the GPR-based residual model, Li et al. [22] conducted an in-depth analysis of the error generation mechanisms in the dynamic model. By reducing the input features of GPR from five dimensions to three, while imposing strict physical constraints on these features, a low-dimensional residual model was constructed. The GPR-based residual modeling paradigm significantly improves the accuracy of vehicle dynamics modeling, thereby enhancing the control performance of high-speed autonomous racing vehicles.

To reduce the computational burden caused by the number of inducing points in GPR, clustering methods are typically employed to partition the dataset [23], with a separate GPR model fitted to each cluster. While this clustering-based GPR residual modeling approach has demonstrated significant effectiveness in data-driven modeling, it still suffers from the following fundamental limitations:

1. High-Dimensional and Nonlinear Characteristics of Vehicle Dynamics. Vehicle dynamics exhibit strong nonlinearities and complex couplings among multiple states, resulting in a high-dimensional and highly correlated feature space. Clustering-based modeling approaches rely heavily on manual feature engineering and predefined similarity metrics, which may overlook latent dependencies and fail to capture the intrinsic structure of nonlinear dynamic behaviors. Furthermore, the need to specify the number of clusters (e.g., the K value in K-means) and the ambiguity of cluster boundaries restricts the generalization capability of these models.
2. Inherent Trade-off Between Modeling Accuracy and Real-time Efficiency. Achieving high prediction accuracy often requires sophisticated models capable of capturing nonlinear and time-varying behaviors. However, such models tend to be computationally expensive and unsuitable for real-time control. Conversely, simplified models offer high computational efficiency but inevitably sacrifice accuracy. This trade-off between precision and real-time performance remains a persistent bottleneck in model-based vehicle motion control frameworks.
3. Information Loss Caused by Feature Reduction. To reduce computational complexity, many existing clustering-based modeling methods apply dimensionality reduction to the input features prior to regression or learning. Such preprocessing inevitably discards critical dynamic information, weakening the model’s ability to represent coupled vehicle dynamics under diverse conditions. The resulting information loss limits both accuracy and adaptability, especially when the model is used for predictive control in complex scenarios.

### 1.3. Contribution

Accurate vehicle dynamics modeling is fundamental to the performance of MPC. However, conventional physics-based models often struggle to balance prediction accuracy with real-time feasibility. To address this issue, this study proposes a hybrid modeling framework that combines a physics-based vehicle dynamics model with a DDR module. The physics-based model serves as the primary prediction structure, while the ensemble learning refinement module is selectively activated based on prediction errors to compensate for unmodeled and time-varying dynamics, thereby improving modeling accuracy without compromising real-time performance. Compared with traditional residual modeling methods based on GPR or clustering, the proposed approach employs an ensemble of lightweight predictors, which eliminates the need for manual clustering and provides stronger nonlinear approximation capability, enhanced robustness, and computational efficiency. The refined dynamics model is directly integrated into the MPC prediction layer, enabling more accurate and robust trajectory tracking across various driving scenarios. The main contributions of this study are summarized as follows:

1. An ensemble learning-based dynamics refinement method is proposed. The ensemble model integrates multiple independently trained predictors to characterize the discrepancy between the nominal model and real vehicle responses. This approach eliminates the need for manual clustering and enhances the adaptability, stability, and accuracy of data-driven dynamic modeling.
2. A feature-driven activation mechanism is developed to adaptively regulate the refinement model. By comparing prediction errors with a predefined threshold, the controller dynamically determines whether refinement is necessary: when errors remain small, refinement is disabled to reduce computational cost; once the threshold is exceeded, the refinement model is activated to enhance predictive accuracy. This strategy effectively balances modeling precision and real-time feasibility.
3. The proposed ensemble learning-enhanced MPC framework is validated through high-fidelity simulations under single lane-change, double lane-change, and slalom (serpentine) scenarios. The results demonstrate that the proposed method significantly improves modeling accuracy, real-time performance, and trajectory tracking precision compared with conventional MPC. In lane-change maneuvers, lateral tracking errors are reduced by approximately 15% and 20%, while the slalom test further verifies the superior stability and robustness of the proposed approach under rapidly varying curvature and highly dynamic steering conditions.

The remainder of this paper is organized as follows: Section 2 presents the construction of the ensemble learning-based dynamics refinement model and describes the corresponding training and activation mechanisms in detail. Section 3 introduces the trajectory tracking control framework, in which the refined dynamic model is integrated into the MPC formulation. Section 4 provides high-fidelity simulation results under single and double lane-change scenarios to validate the effectiveness of the proposed approach. Finally, Section 5 concludes the paper and discusses future research directions.

## 2. Vehicle Dynamics Model

**Figure 1:**
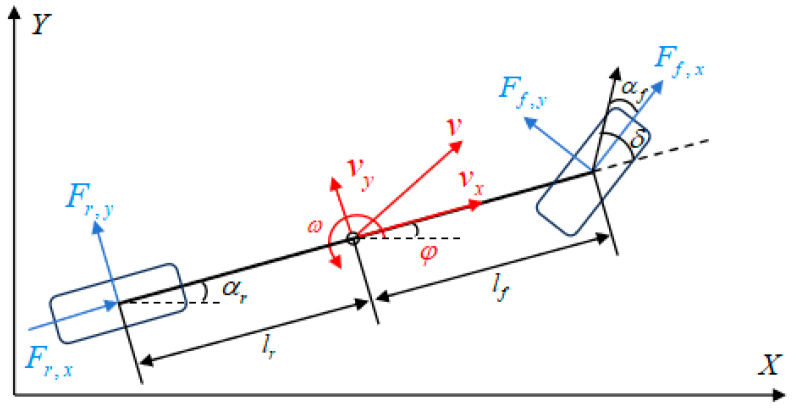
Three-degree-of-freedom single-track vehicle dynamic model.

The established vehicle dynamics model is shown in Figure 1. This three-degree-of-freedom (3-DOF) model is a widely adopted and well-validated framework in the vehicle dynamics and control literature, used to describe the longitudinal, lateral, and yaw motions of the vehicle. The state variables of the model are as follows:

(1) $$x={\left[X,Y,\varphi ,v_{x},v_{y},\omega ,\delta \right]}^{T}.$$

where $p={\left[X,Y\right]}^{T}$ denotes the vehicle position, and ϕ represents the heading angle. $v_{x}$ and $v_{y}$ are the longitudinal and lateral velocities, respectively, and ω is the yaw rate. These states are measured or estimated using onboard pose sensors, such as GNSS/INS and inertial sensors. The front wheel steering angle is denoted by δ, which is obtained from steering sensors. The control input is defined as u=Δδ, representing the variation in the steering angle. Here, the state variables are defined in the global (inertial) coordinate frame, which is defined as a right-handed Cartesian coordinate system in the horizontal plane. The X- and Y-axes represent the global longitudinal and lateral directions, respectively.

We construct vehicle dynamics using the Dynamics with Data-driven Refinement (DDR) model, which augments a physics-based analytical formulation with an additional compensation term learned through an ensemble learning framework. The analytical component provides the structured representation of the vehicle dynamics, while the ensemble-based refinement captures nonlinear and time-varying behaviors that are difficult to express explicitly. Together, these components form the DDR model, which improves the fidelity of the overall dynamic representation.

(2) $$\dot{x}=f(x,u)=f_{ana}(x,u)+\Gamma_{k}g_{ENS}(z)$$

(3) $$z=B_{z}{\left[x^{T}\;u^{T}\right]}^{T}$$

where $f_{ana}$ represents the analytical dynamics, $g_{ENS}$ the ensemble learning-based compensation model, z is the feature vector formed from states x and inputs u via the feature-selection matrix $B_{z}$, and $\Gamma_{k}$ via a component-wise activation matrix determining which states are refined at time step k.

This formulation ensures that the DDR model retains the computational efficiency and interpretability of the analytical dynamics while benefiting from the adaptive refinement capability provided by ensemble learning.

### 2.1. Refinement Activation Rule

When the vehicle dynamics operate within the linear region, the contribution of the refinement model is typically small. In such cases, the compensation mechanism yields only marginal improvement, as the refined prediction becomes nearly identical to that of the analytical model, while unnecessarily increasing computational cost.

To address the aforementioned issue, we propose a conditional activation strategy based on dynamic characteristics. In this framework, the analytical dynamics are employed as an integrator, where the measured state $x_{k}$ of the vehicle at time step k is combined with the optimal control input $u_{k}$ computed by the MPC to predict the next-step state ${\hat{x}}_{k+1}$. Subsequently, the prediction error $e_{k+1}$ is calculated as the difference between the predicted state ${\hat{x}}_{k+1}$ and the measured state $x_{k+1}$ obtained from sensors at time step k+1. The data-driven refinement is then applied in a component-wise manner. We define a diagonal gating matrix $\Gamma_{k}\;=\;\mathrm{diag}(\gamma_{v_{y},k},\gamma_{\omega ,k})$, where

(4) $$\gamma_{v_{y},k}=H\left(\left|e_{v_{y},k}\right|-\tau_{v}\right),\;\gamma_{\omega ,k}=H\left(\left|e_{\omega ,k}\right|-\tau_{\omega }\right)$$

and $H\left(•\right)$ denotes the Heaviside step function defined as

$$H(x)=\left\{\begin{array}{c} 1,\;x>0\\ 0,\;x\leq 0 \end{array}\right.$$

Here, $\tau_{v_{y}}$ and $\tau_{\omega }$ predefined activation thresholds for the lateral velocity-related and yaw-rate-related components, respectively. This activation rule enables the refinement model to operate selectively by turning on compensation only when the prediction error exceeds the threshold. Consequently, redundant computations are avoided during benign operating conditions, while the model’s adaptability is enhanced under nonlinear or time-varying dynamics, providing a more efficient and robust refinement mechanism for complex vehicle systems.

### 2.2. Analytical Dynamic Model

In this study, the analytical dynamic model is derived by comprehensively considering both the kinematic relationships and the dynamic characteristics of the vehicle. Based on the single-track assumption, a three-degree-of-freedom (3-DOF) model is formulated to capture the longitudinal, lateral, and yaw motions of the vehicle. The model integrates the effects of lateral tire forces, yaw moment, and vehicle motion constraints to accurately describe the vehicle’s dynamic behavior. The structure and variables of the model are illustrated in Figure 1. The model expression is given as follows:

(5) $$\dot{x}=\left[\begin{array}{c} v_{x}\cos\varphi -v_{y}\sin\varphi \\ v_{x}\sin\varphi +v_{y}\cos\varphi \\ \omega \\ \frac{1}{m}\left(F_{xr}-F_{yf}\sin\delta +F_{xf}\cos\delta \right)+v_{y}\omega \\ \frac{1}{m}\left(F_{yr}+F_{yf}\cos\delta +F_{xf}\sin\delta \right)-v_{x}\omega \\ \frac{1}{I_{z}}\left((F_{yf}\cos\delta +F_{xf}\sin\delta )l_{f}-F_{r,y}l_{r}\right)\\ \dot{\delta } \end{array}\right]$$

where $F_{xf}$ and $F_{xr}$ represent the longitudinal forces exerted by the tires on the front and rear axles, respectively, while $F_{yf}$ and $F_{yr}$ correspond to the lateral forces on the front and rear tires. The variable m denotes the mass of the vehicle, $I_{z}$ refers to the vehicle’s yaw moment of inertia, and $l_{f}$, $l_{r}$ are the distances from the vehicle’s center of gravity to the front and rear axles, respectively. The tire forces $F_{xf}$, $F_{xr}$, $F_{yf}$, and $F_{yr}$ are modeled using the widely adopted simplified Magic Formula (Pacejka) model [24], which characterizes the nonlinear mapping from slip ratio λ and slip angle α to longitudinal and lateral tire forces.

(6) $$\alpha_{f}=\arctan\left(\frac{v_{y}+l_{f}\omega }{v_{x}}\right)-\delta$$

(7) $$\alpha_{r}=\arctan\left(\frac{v_{y}-l_{r}\omega }{v_{x}}\right)$$

(8) $$\lambda =\left\{\begin{array}{c} \frac{nR-v_{x}}{nR},v_{x}<nR\\ \frac{v_{x}-nR}{v_{x}},v_{x}\geq nR \end{array}\right.$$

where n denotes the wheel rotational speed, and the Magic Formula is given by the following:

(9) $$Y(x)=D\sin\left\{C\arctan\left[Bx-E(Bx-\arctan(Bx)))\right]\right\}$$

where B, C, D, and E are parameters of the Magic Formula following the fitting process, and the parameters are presented in Table 1.

### 2.3. DDR Model

**Figure 2:**
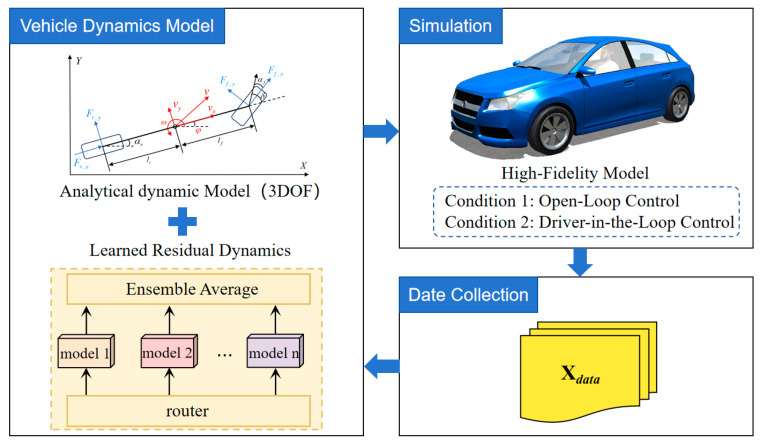
Data-Driven Dynamics Refinement with $f_{\mathrm{DDR}}=f_{\mathrm{ANA}}+f_{\mathrm{LRD}}$: data are collected from a high-fidelity vehicle model under multiple operating conditions, including open-loop control and driver-in-the-loop control. The analytical dynamics model (ANA) is augmented by a learned residual dynamics model (LRD), which is trained on high-fidelity data using an ensemble learning strategy. The resulting data-driven dynamics refinement (DDR) is then integrated into the MPC framework to achieve accurate and robust trajectory tracking.

To enhance the predictive capability of the analytical dynamic model and improve the overall performance of MPC, this study develops a data-driven dynamics refinement framework based on an ensemble learning architecture. As illustrated in Figure 2, the workflow consists of three main stages: (i) data collection and preprocessing using a high-fidelity simulation platform; (ii) training the ensemble learning-based refinement model with the processed dataset; and (iii) validating and fine-tuning the trained model through iterative optimization to ensure robust performance under diverse driving conditions.

#### 2.3.1. Data Collection and Processing

A high-fidelity vehicle dynamics model is established in a CarSim–Simulink (CarSim 2022.1; Simulink R2023a) co-simulation environment. Open-loop simulation data are collected at 10 Hz under a wide range of speed and steering inputs to cover representative driving scenarios. In addition, controller-in-the-loop simulations are conducted, where an MPC executes trajectory tracking tasks to generate closed-loop data for model learning. All collected data are filtered, normalized, and synchronized to form the final dataset for training, comprising approximately 2.6 GB of recordings.

#### 2.3.2. Model Training

To establish the mapping between the system state–input pair and the dynamics refinement term, the ensemble refinement model is trained using supervised learning. The sampling period is defined as $\Delta t=t_{k+1}-t_{k}$. Given $\left(x_{k},u_{k}\right)$ and using the analytical dynamic model as the state integrator, the one-step prediction is

(10) $${\hat{x}}_{k+1}=x_{k}+\Delta tf_{ana}(x_{k},u_{k})$$

The refinement target is computed by comparing the predicted state with the sensor-derived state at the next time step. Specifically, the correction term is defined as

(11) $$y_{e,k}=\frac{x_{k+1}-{\hat{x}}_{k+1}}{\Delta t}+w_{k}$$

where $w_{k}$ denotes the stochastic disturbance term. The input consists of the state vector $x_{k}$ and the control input $u_{k}$, while the supervisory signal corresponds to the residual output $y_{e,k}$. Through supervised regression, the ensemble learning framework establishes a nonlinear mapping

$$g_{ENS}:z_{k}\mapsto y_{k}$$

which enables the model to capture unmodeled dynamics, time-varying behaviors, and systematic prediction biases of the analytical model. To improve robustness and generalization, the ensemble model integrates M lightweight base learners. Each learner independently estimates a refinement component, and the final output is obtained by averaging the predictions:

$$g_{ENS}\left(z_{k}\right)=\frac{1}{M}\sum_{i=1}^{M}g_{i}\left(z_{k}\right)$$

Each base learner $g_{ENS}\left(•\right)$ is implemented as a shallow multilayer perceptron (MLP) to ensure fast inference. All input features are standardized using z-scores, and the training target corresponds to the normalized one-step refinement term $y_{k}$.

The ensemble model is trained using a supervised regression loss formulated as follows:

$$L=\sum B_{d}g_{ENS}\left(z_{k}\right)-{y_{k}}_{2}^{2}+\lambda_{wd}\|\theta {\|}_{2}^{2}$$

where θ denotes all learnable parameters of the ensemble, and $\lambda_{wd}$ is a weight-decay coefficient used to improve generalization and prevent overfitting.

**Figure 3:**
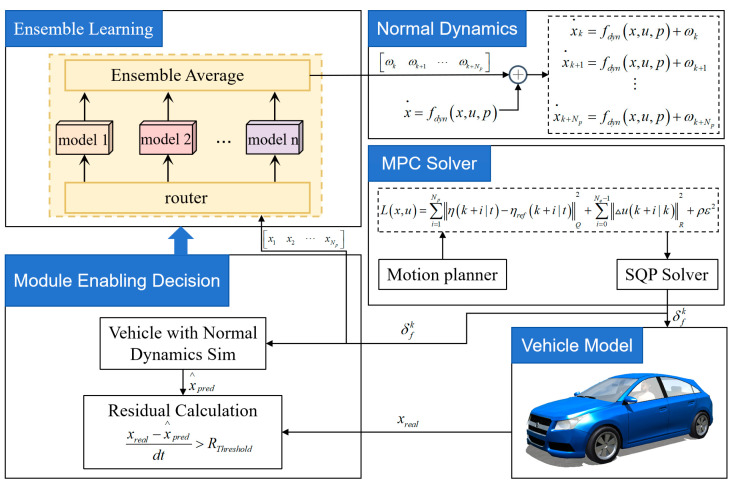
Ensemble-Enhanced MPC framework.

After training, the ensemble-based refinement model is evaluated under representative driving scenarios to assess its prediction accuracy, generalization capability, and real-time computational performance. The validation focuses on the convergence of the refinement term, the consistency between refined and measured dynamics, and the stability of the ensemble output across varying operating conditions. The overall framework and computational procedure of the refinement-enhanced MPC are summarized in Figure 3 and Algorithm 1.
**Algorithm 1** Ensemble-Enhanced MPC Algorithm$\mathrm{Require}:\;\mathrm{Current}\;\mathrm{state}\;x_{k}$$;\;\mathrm{lateral}\;\mathrm{velocity}\;\mathrm{error}\;\mathrm{threshold}\;e_{v_{y}}$$;\;\mathrm{yaw}-\mathrm{rate}\;\mathrm{error}\;\mathrm{threshold}\;e_{\omega ,t}$$\mathrm{Ensure}:\;\mathrm{Optimal}\;\mathrm{control}\;\mathrm{input}u_{k}$1:$\mathrm{If}\;\overset{\wedge }{x_{k}}$ is uninitialized **then**2:$\overset{\wedge }{x_{k}}\leftarrow 0$3:**end if**4:**Prediction errors:**5:$\begin{array}{l} e_{v_{y},k}\leftarrow \frac{x_{v_{y},k}-{\hat{x}}_{v_{y},k}}{\Delta t},\\ e_{\omega ,k}\leftarrow \frac{x_{\omega ,k}-{\hat{x}}_{\omega ,k}}{\Delta t} \end{array}$6:$\mathrm{Compute}\;\gamma_{\omega ,k}$$\;\mathrm{and}\;\gamma_{v_{y},k}$ by (4).7:Update the MPC prediction equations by (2).8:Set the MPC reference trajectory using the multi-point tracking method.9:$\mathrm{Solve}\;\mathrm{the}\;\mathrm{MPC}\;\mathrm{to}\;\mathrm{obtain}\;\mathrm{the}\;\mathrm{optimal}\;\mathrm{control}\;u_{k}$.10:**State propagation:**11:${\hat{x}}_{k+1}\leftarrow x_{k}+\Delta tf_{ana}(x_{k},u_{k})$12:k←k+1

## 3. Vehicle Motion Control Framework

### 3.1. Lateral MPC with Ensemble-Based Dynamic Refinement

In general terms, MPC adjusts the control action by minimizing a cost function L(x,u) over a receding prediction horizon, subject to the system dynamics constraints $\dot{x}=f(x,u)$, in order to ensure that the system output closely follows the reference states $x_{r}(t)$ and control inputs $u_{r}(t)$:

(12) $$\begin{array}{l} \underset{u}{min}\int L(x,u)\\ s.t.\dot{x}=f(x,u)\\ x(t_{0})=x_{0}\\ r(x,u)=0\\ h(x,u)>0 \end{array}$$

where $x_{0}$ represents the initial conditions, and h and r are the inequality and equality constraints, respectively. In MPC, the cost function $L\left(x,u\right)$ is most commonly quadratic, as this form enhances the numerical stability of the optimization process. The system is discretized over the prediction horizon T into N steps, with a step size of Δt=T/N. Additionally, during the MPC optimization process. It is essential to impose appropriate constraints $u_{\min}\leq u_{k}\leq u_{\max}$ to satisfy the physical limitations of the actuators. Ultimately, the activation rule determines whether the refinement term produced by the ensemble learning model should be incorporated into the dynamic constraints. If the activation condition is not satisfied, the controller relies solely on the analytical dynamic model. Conversely, if the residual rule is triggered, the state sequence $\left[\begin{array}{cccc} x_{1} & x_{2} & \cdots & x_{Np} \end{array}\right]$ is first computed using MPC. This state sequence is then input into the model to obtain the predicted residual sequence $\left[\begin{array}{cccc} \omega_{1} & \omega_{2} & \cdots & \omega_{Np} \end{array}\right]$, which then imposes the DDR dynamics as constraints over the subsequent $N_{p}$ steps.

(13) $$\begin{array}{c} {\dot{x}}_{k}=f(x,u)+\omega_{1}\\ {\dot{x}}_{k+1}=f(x,u)+\omega_{2}\\ \vdots \\ {\dot{x}}_{k+Np}=f(x,u)+\omega_{Np} \end{array}$$

The system states are discretized using the fourth-order Runge–Kutta method, after which the MPC objective function is expressed in discrete-time form

(14) $$\begin{array}{l} \underset{u_{k}}{\min}\sum_{k=0}^{N}({\left(y_{k}-y_{r,k}\right)}^{T}Q(y_{k}-y_{r,k})+{\left(u_{k}-u_{r,k}\right)}^{T}R_{1}(u_{k}-u_{r,k}))\\ s.t.\;x_{k+1}=f_{RK4}(x_{k},u_{k},\Delta t)\\ x_{k}=x_{0}\\ u_{\min}\leq u_{k}\leq u_{\max} \end{array}$$

In this formulation, the control input represents the increment of the front-wheel steering angle, ensuring smooth steering behavior and avoiding abrupt changes in the steering command. The objective function penalizes both the tracking error and the control effort, guiding the vehicle to follow the reference trajectory while maintaining steering smoothness.

To define the physical constraints of the steering actuator, we conducted a dedicated vehicle steering test. The front-wheel angle and its rate of change were recorded under normal operating conditions, and the averaged results were used to determine the admissible bounds. Based on experimental data, the maximum steering angle is limited to 30°, and the allowable increment of the steering angle per control step is constrained to 0.47°. These limits are incorporated into the MPC optimization as input constraints to ensure that the generated control commands remain feasible for the real steering mechanism.

In solving the MPC, the reference control input within the prediction horizon is initialized with the control value from the previous step, and the reference state variables $y_{r,k}=\left[\begin{array}{ccc} X_{r,k} & Y_{r,k} & \varphi_{r,k} \end{array}\right]\;(k=1,2,\ldots ,N)$ are generated through a multi-point tracking method. Specifically, the reference state is determined based on the current longitudinal speed $v_{x}$ and the discrete time step. The theoretical travel distance for a single time step is calculated as $s=v_{x}\cdot \Delta t$. A real-time trajectory projection is employed to find the matching point $P_{d}$ on the reference path corresponding to the vehicle’s look-ahead control point:

$$P_{e}\;=\;\left[\begin{array}{c} X\\ Y \end{array}\right]\;+\;l_{s}\left[\begin{array}{c} \cos\varphi \\ \sin\varphi \end{array}\right],$$

where $l_{s}$ denotes the look-ahead distance, the corresponding state values X, Y, and φ are then taken as the reference state for the first step of the prediction horizon $y_{r1}$. To construct the complete reference sequence, the number of trajectory point jumps $n=\left\lfloor \frac{s}{\delta_{s}}\right\rfloor$ is calculated based on the discretization step size of the reference trajectory, where $\left\lfloor \cdot \right\rfloor$ denotes the floor operator to ensure valid indexing, $\delta_{s}$ denotes the arc-length spacing between adjacent points on the reference trajectory. Starting from the matching point $P_{d}$, the reference states for the subsequent N steps are sequentially extracted along the reference trajectory with a step size of n, forming the reference sequence. Multi-point tracking enables the controller to consider the upcoming curves along the path, allowing for more effective adjustment of the control strategy. Using multi-point preview enables the controller to anticipate upcoming curvature and heading changes within the prediction horizon, improving tracking accuracy.

To efficiently solve this quadratic optimization problem, a multiple shooting method is employed to formulate the problem model, and the Sequential Quadratic Programming (SQP) method is used for real-time iterative solving. All implementations are carried out based on the high-performance computing frameworks ACADOS (v0.2.5) [25] and CasADi (v3.3.5) [26].

### 3.2. Feedforward Augmentation

Feedforward control predicts and adjusts the system output based on known system input information, effectively reducing system errors. When the curvature of the reference trajectory is large, feedforward control can respond proactively, mitigating overshoot and bringing the output signal closer to the desired value. Due to the inherent understeering characteristics in vehicle design, such as high-speed operation, changes in wheel toe, and elastic deformations of the steering system, the understeering behavior becomes more pronounced. Vehicle understeering can be represented as follows:

(15) $$kus=\frac{m\cdot lr}{K_{f}\cdot l}-\frac{m\cdot lf}{K_{r}\cdot l}$$

where l is the wheelbase of the vehicle; $K_{f}$ is the front axle cornering stiffness; and $K_{r}$ is the rear axle cornering stiffness. The nonlinear feedforward control law is designed based on the curvature of the matching point corresponding to the vehicle’s lookahead point:

(16) $$\delta_{ff}=l\cdot \rho +kus\cdot v_{x}^{2}\cdot \rho$$

where $\delta_{ff}$ represents the feedforward steering angle, and ρ denotes the curvature of the matching point. Ultimately, the feedforward steering angle computed by the feedforward algorithm is combined with the steering angle obtained from the MPC to form the final steering command, which is then input into the actuator to perform the corresponding steering operation.

## 4. Simulation Results and Analysis

**Figure 4:**
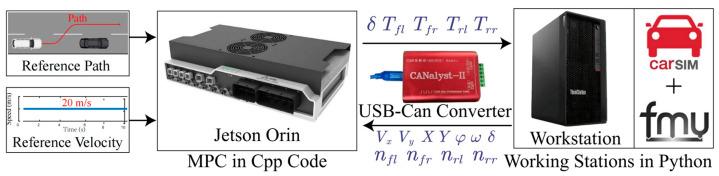
Ensemble-enhanced MPC: an activation rule adaptively determines whether the analytical dynamic model is used alone or augmented with the refinement model during trajectory prediction.

This study develops a high-fidelity validation environment by integrating the CarSim-Python co-simulation platform with real-time control systems. A vehicle simulation model is built in CarSim, and the key parameters of this model are provided in Table 2. All vehicle parameter values are factory-calibrated and used to construct a high-fidelity vehicle model. They represent the intrinsic physical properties of the vehicle and therefore do not vary with weather or road conditions. Within this environment, the Industrial PC (IPC) executes the proposed control strategy and facilitates data exchange with the CarSim FMU interface on a PC, where dynamic simulations are carried out using Python (v3.9.13). A USB-CAN interface enables bidirectional CAN signal communication between the IPC and a secondary PC running Python. Acados is employed to generate C++ code, serving as a fast embedded development framework with efficient nonlinear optimization solving capabilities based on the HPIPM algorithm. This allows rapid deployment and real-time execution of the control algorithm. In addition, the ensemble learning model is accelerated using TensorRT (v8.6.1) to ensure low-latency inference during closed-loop simulations. The overall workflow, illustrated in Figure 4, establishes a reliable and efficient experimental environment for validating advanced and computationally demanding control strategies.

### 4.1. Ensemble Model Training and Evaluation

**Figure 5:**
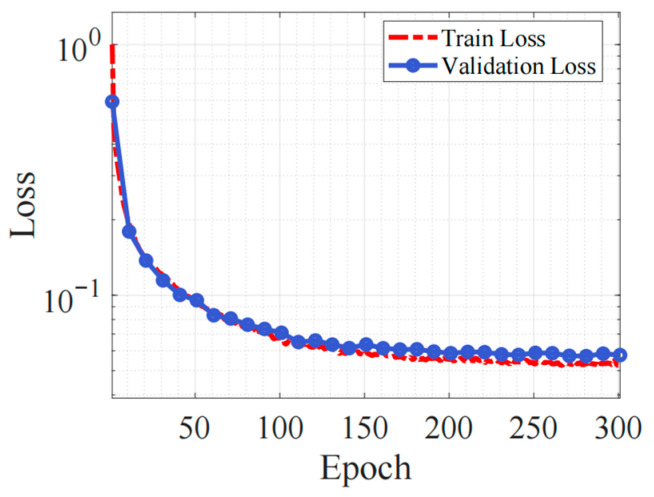
Residual Dynamics Training for DDR Model.

The ensemble-based refinement model is trained using the collected offline simulation data. The model training and evaluation pipeline is developed based on the PyTorch (v1.13.1)deep learning framework. The Adam optimizer is employed with an initial learning rate of 0.001, while an exponential decay learning rate scheduler (decay factor of 0.98) is used to gradually reduce the learning rate and improve model stability. In the ensemble architecture, multiple lightweight base learners are trained jointly, and their outputs are averaged to produce the final refinement term. The dataset used for the experiments is split into 70% for training and 30% for testing. The loaded dataset is standardized to ensure that input features have zero mean and unit variance, enhancing the model’s convergence stability. The training is conducted on an NVIDIA RTX 4090 GPU with 300 training epochs. During training, the model is evaluated on the test set every 10 epochs, and the average loss is recorded. The training results are shown in Figure 5.

**Figure 6:**
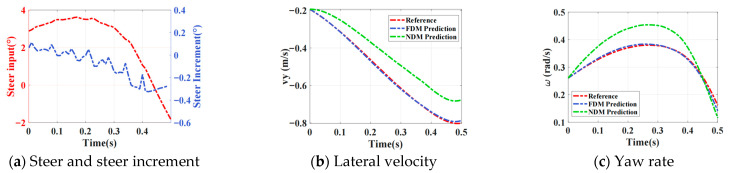

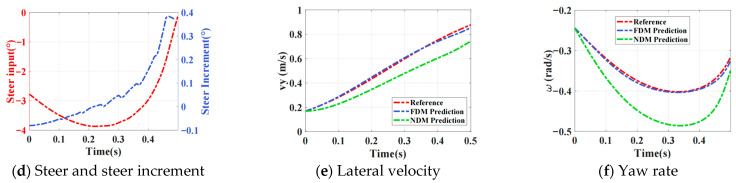
Under Identical Inputs: DDR Model and ANA Model Predictions vs. Reference.

Figure 6 shows validation on the collected test dataset: rolling integration predictions of vehicle states over a 0.5 s horizon, given random initial conditions and varying control inputs. In this study, the MPC variable is defined as the front-wheel steering angle increment. Figure 6a,d present the variations in control inputs and the corresponding front-wheel steering angles. Figure 6b,e show that, after incorporating the ensemble-based refinement model, the analytical dynamic model achieves a substantial improvement in the prediction accuracy of the vehicle’s lateral velocity. Similarly, Figure 6c,f demonstrate that the refined model also provides more accurate yaw-rate predictions compared with the uncompensated analytical model. The quantitative results are summarized in Table 3.

### 4.2. Real-Time Control Strategy Validation Through Co-Simulation

At relatively high vehicle speeds, the mismatch between nominal physics-based vehicle dynamics models and actual vehicle behavior tends to become more pronounced, particularly in large-curvature maneuvers such as single and double lane-change scenarios. Therefore, a reference speed of 72 km/h is selected in this study to sufficiently expose model uncertainties and to more clearly evaluate the effectiveness of the proposed DDR model in compensating for modeling errors under high-speed and highly dynamic conditions.

Meanwhile, to further enhance the completeness of the comparison and to systematically assess the robustness of the proposed method across different operating conditions, a slalom test at 50 km/h is also designed. Although the vehicle speed in this scenario is relatively lower, the frequent steering reversals and pronounced dynamic characteristics impose more stringent requirements on the transient response and predictive accuracy of the control system, thereby providing a strong and complementary validation to the high-speed lane-change maneuvers.

#### 4.2.1. Double-Lane Change Test

**Figure 7:**
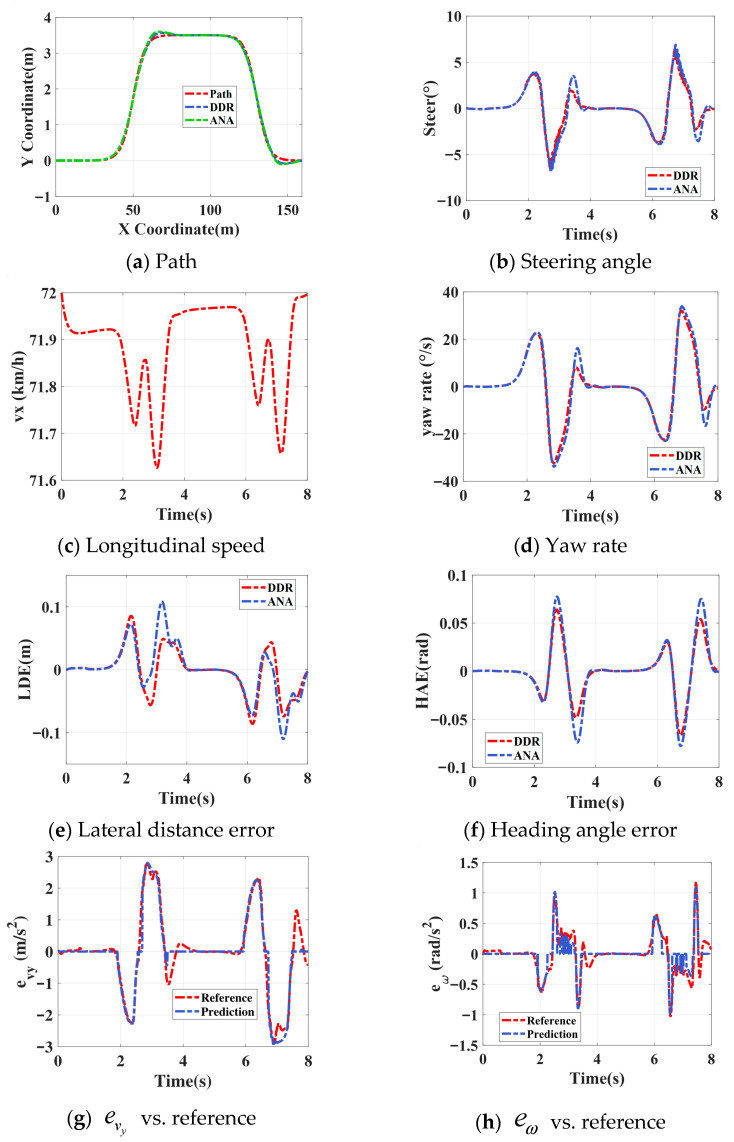
Double-lane change results.

Figure 7 presents the trajectory tracking results under a double-lane-change scenario at a target speed of 72 km/h and a road adhesion coefficient of 0.9. As shown in Figure 7a, both models follow the reference path well, while the refined model exhibits faster and more accurate responses in curved segments. The steering input in Figure 7b is smoother, avoiding high-frequency oscillations. From Figure 7c,d, both controllers maintain stable longitudinal velocity; however, the refined model achieves a smaller overshoot and faster convergence in yaw rate. As seen in Figure 7e,f, the refined model reduces the maximum lateral deviation by approximately 6 cm and decreases the heading error by about 0.02 rad, indicating improved tracking accuracy. Furthermore, Figure 7g,h demonstrate that the refined model predicts lateral and yaw dynamics more consistently with the reference, enabling timely correction of dynamic errors and enhancing overall vehicle stability. Overall, the proposed MPC framework with ensemble-based dynamic refinement achieves superior path-tracking accuracy, smoother control actions, and enhanced dynamic stability compared with the nominal model.

#### 4.2.2. Single-Lane Change Test

**Figure 8:**
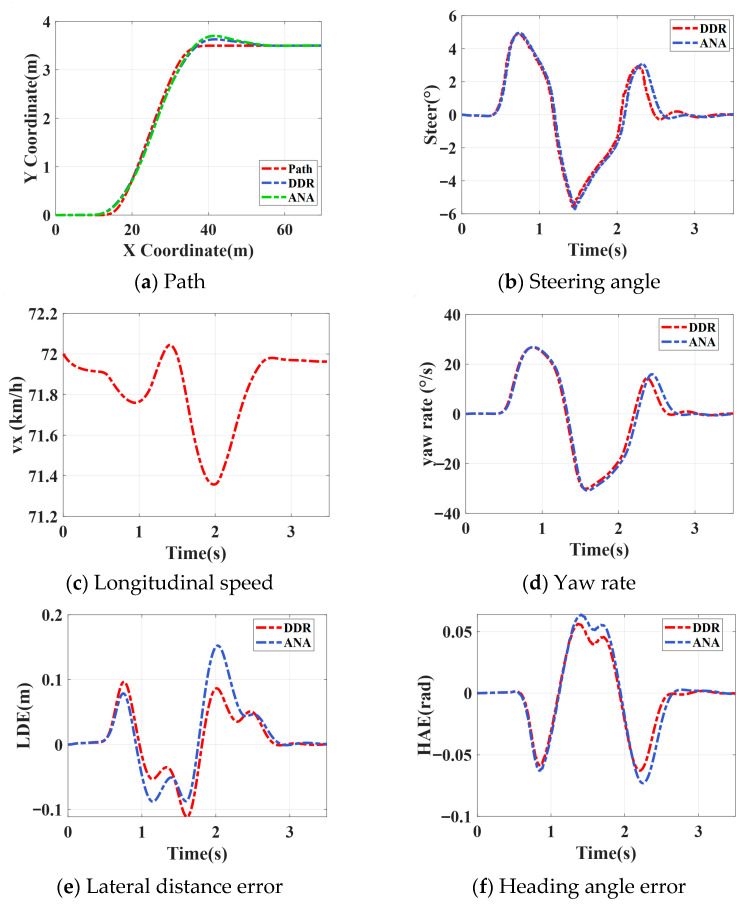

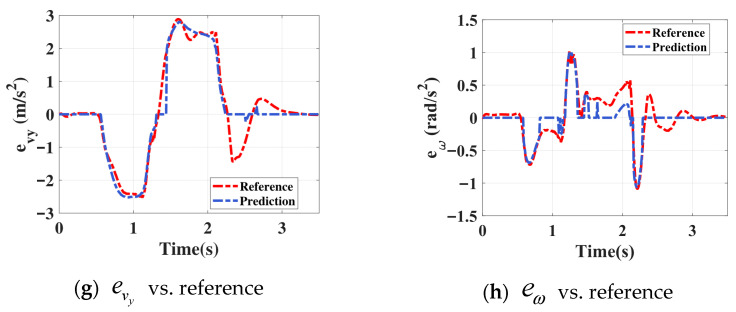
Single-lane change results.

Figure 8 illustrates the path-tracking performance under a single-lane-change maneuver at a reference speed of 72 km/h and a road adhesion coefficient of 0.9. As shown in Figure 8a, both control models successfully follow the planned trajectory, while the refined model exhibits higher responsiveness in regions with large steering variations. In Figure 8b, the refined model generates smoother steering inputs and effectively suppresses oscillatory behavior. From Figure 8c,d, both models maintain stable longitudinal velocity, but the refined model produces a yaw-rate response that aligns more closely with the reference and shows reduced overshoot. Figure 8e,f reveal that the refined model achieves smaller lateral and heading errors, reducing the maximum lateral deviation by about 4 cm and the maximum vehicle heading error by approximately 0.015 rad. Furthermore, as seen in Figure 8g,h, the refined model more accurately captures the variations in lateral and yaw dynamics, thereby improving prediction precision and overall dynamic consistency of the control system. The training and testing losses of the ensemble-based refinement model eventually converge, indicating that the network rapidly learns effective feature representations. Moreover, the small gap between the testing and training losses, without any noticeable overshoot, demonstrates that the model possesses strong generalization capability.

#### 4.2.3. Slalom Test

**Figure 9:**
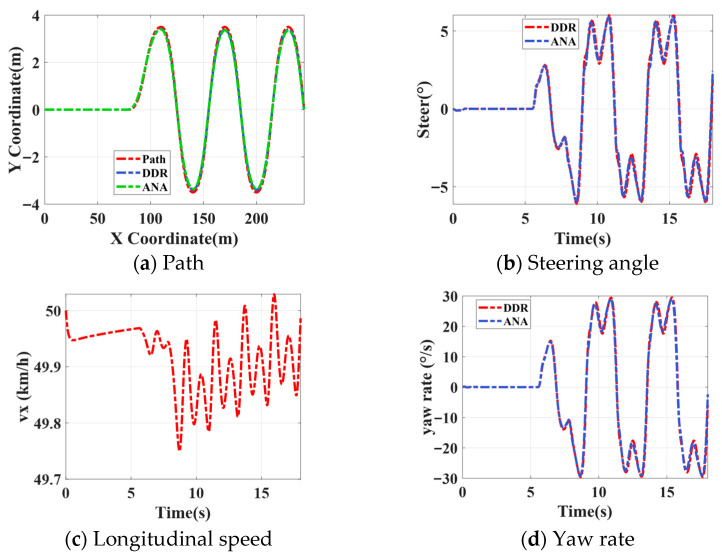

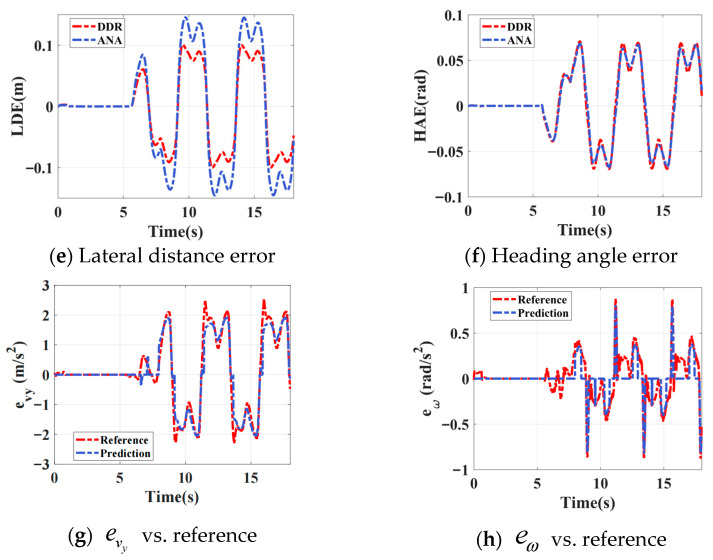
Slalom results.

A serpentine path-tracking experiment is conducted at a constant speed of 50 km/h to evaluate controller performance under rapidly varying curvature conditions. As shown in Figure 9a, both DDR and ANA are able to stably follow the reference path without trajectory divergence, while the longitudinal speed remains well-regulated throughout the maneuver Figure 9c. From the error perspective, Figure 9e,f present the lateral displacement error and heading angle error, respectively. Compared with ANA, DDR exhibits smaller error amplitudes during peak steering phases, with the peak LDE maintained within approximately 0.1 m, indicating improved tracking accuracy under high-frequency curvature transitions. Further analysis of Figure 9b,d show that DDR achieves this error reduction without introducing more aggressive control actions, as evidenced by smoother yaw rate and steering angle responses. Finally, the close agreement between the predicted and reference yaw and lateral accelerations in Figure 9g,h confirm that DDR maintains higher dynamic prediction fidelity under the serpentine maneuver, thereby contributing to improved tracking stability and robustness.

## 5. Conclusions

This study presents an enhanced MPC framework that integrates an ensemble learning-based DDR model to address fundamental limitations of conventional physics-based vehicle dynamics modeling in high-dynamic driving scenarios. By retaining a nominal physics-based model as the structural backbone and augmenting it with data-driven residual dynamics learning, the proposed framework achieves a favorable balance between modeling accuracy, interpretability, and real-time feasibility.

Methodologically, the vehicle dynamics are explicitly decomposed into nominal and refinement components, enabling the proposed DDR model to effectively capture high-dimensional, nonlinear, and time-varying effects arising from tire–road interactions, load transfer, and parameter uncertainties. Compared with learning-based MPC approaches that rely on Gaussian processes, clustering strategies, or fully data-driven models, the ensemble learning-based DDR framework provides a scalable and computationally efficient alternative without requiring manual feature engineering or predefined operating-condition partitions. Furthermore, the proposed feature-driven activation mechanism selectively enables refinement of dynamics only when significant modeling discrepancies are detected, thereby mitigating unnecessary computational burden and enhancing suitability for real-time control applications.

From an application perspective, extensive high-fidelity simulations under representative high-dynamic driving conditions—including single lane-change, double lane-change, and slalom maneuvers—demonstrate that the proposed DDR-enhanced MPC consistently outperforms traditional MPC. The results show improved model prediction accuracy, smoother control actions, enhanced trajectory tracking performance, and increased robustness across different speeds and maneuver types. These findings indicate that the proposed approach effectively enhances the reliability of MPC in sharp-curvature and rapidly changing driving scenarios, which are critical for real-world intelligent and autonomous vehicle deployment.

Overall, this work contributes a unified, implementable, and extensible learning-augmented MPC solution that bridges the gap between theoretical data-driven modeling and practical vehicle motion control. Future research will focus on extending the framework to coupled longitudinal–lateral control, as well as incorporating online adaptive learning mechanisms to further improve robustness and generalization under more complex and uncertain operating conditions.

## Figures and Tables

**Table 1:** Fitted Magic Formula parameters for longitudinal and lateral forces.

Direction	B	C	D	E
Longitudinal force ($F_{x}$)	−0.203	0.978	3513.520	−9.620
Lateral force ($F_{y}$)	13.59	−2.093	−4206.900	−1.218

**Table 2:** Vehicle parameter values used in the simulation.

Parameter	Value	Parameter	Value
Vehicle curb weight (kg)	1360	Moment of inertia about the *Z*-axis (kg · m^2^)	1805
Wheelbase (m)	2.035	Track width (mm)	1356 (front, rear)
Distance from the center of gravity to the front axle (m)	1.117	Distance from the center of gravity to the rear axle (m)	1.188
Center of gravity height (m)	0.525	Rolling radius of the wheel (m)	0.283
Front axle cornering stiffness (N/rad)	105,991	Rear axle cornering stiffness (N/rad)	106,456

**Table 3:** Comparison of DDR and ANA prediction errors vs. reference under identical inputs.

		DDR Prediction vs. Reference		ANA Prediction vs. Reference	
		Average	RMSE	Average	RMSE
Test 1	$v_{y}$	0.0100	0.0125	0.0973	0.1071
	ω	0.0038	0.0045	0.0627	0.0670
Test 2	$v_{y}$	0.0042	0.0053	0.0935	0.1001
	ω	0.0046	0.0063	0.0486	0.0573

## Data Availability

The original contributions presented in this study are included in the article. Further inquiries can be directed to the corresponding author.
